# Strongyloides stercoralis Infection in Humans: A Narrative Review of the Most Neglected Parasitic Disease

**DOI:** 10.7759/cureus.46908

**Published:** 2023-10-12

**Authors:** Mary Y Yeh, Sanjana Aggarwal, Margaret Carrig, Ahad Azeem, Anny Nguyen, Shannon Devries, Chris Destache, Toan Nguyen, Manasa Velagapudi

**Affiliations:** 1 Internal Medicine, Creighton University School of Medicine, Omaha, USA; 2 Medicine, Hamdard Institute of Medical Sciences and Research, New Delhi, IND; 3 Infectious Diseases, Creighton University School of Medicine, Omaha, USA; 4 Internal Medicine, Creighton University, Omaha, USA; 5 Pharmacy Practice, Creighton University School of Pharmacy and Health Professions, Omaha, USA; 6 Internal Medicine, Houston Methodist Hospital, Houston, USA

**Keywords:** covid-19, larva currens, disseminated infection, hyperinfection syndrome, strongyloidiasis

## Abstract

Strongyloidiasis is a helminth infection affecting 613.9 million people annually, mainly in the tropics and subtropics. The reported seroprevalence in the United States is 4% with most of the cases reported in immigrants. Human T-lympho-tropic virus 1 (HTLV-1) infections, hypogammaglobulinemia, immunosuppressant use - particularly steroid use, alcoholism, and malnutrition have been associated with an increased risk of strongyloidiasis. Recently, cases of strongyloidiasis hyperinfection syndrome have been described in coronavirus disease 2019 (COVID-19) patients treated with steroids as well.

This brief review discusses the epidemiology, clinical features, management, and prevention of strongyloidiasis including some facts about the infection in pregnancy, transplant recipients, and COVID-19 patients. We conducted an online search using the PubMed, Scopus, and Google Scholar databases.

Strongyloidiasis can be asymptomatic or present with mild symptoms. *Strongyloides stercoralis* is known to cause autoinfection. In immunocompromised individuals, it can present with severe symptoms, hyperinfection, or disseminated disease. Reported mortality in cases of disseminated Strongyloidiasis is 87.1%. Serology and detection of larvae in stool by direct microscopy are the most commonly used methods to diagnose strongyloidiasis. The drug of choice for the treatment is ivermectin. However, the use of ivermectin in human pregnancy is not well studied, and its teratogenic risks are unknown. Proactive screening of strongyloidiasis is necessary in immunocompromised individuals to prevent severe disease.

## Introduction and background

Strongyloidiasis is a helminth infection most commonly caused by the intestinal nematode *Strongyloides stercoralis* (*S. stercoralis*) and less commonly by *S. fuelleborni*. Previous reports estimate *S. stercoralis*, which is endemic to tropical and subtropical areas, to be responsible for 30-100 million infections annually [1-3]. However, with the development of more sensitive tests, it is now estimated that 613.9 million people annually are infected with *S. stercoralis*, with a vast majority concentrated in Southeast Asia, Africa, and the Western Pacific area [4]. 

In this review, the epidemiology, clinical features, management, prevention of strongyloidiasis, and facts about the infection in pregnancy, transplant recipients, and COVID-19 patients are discussed.

Methods

Papers published through March 2023 from PubMed, Scopus, and Google Scholar databases were retrieved with the terms “Strongyloidiasis,” “Strongyloidiasis in COVID-19,” “Hyperinfection,” “Strongyloidiasis in transplant patients,” and “Strongyloidiasis in transplant.” We included content from case reports, review articles and research articles.

## Review

Epidemiology

Research conducted on immigrant populations has revealed a significantly higher percentage of individuals infected with *Strongyloides*, ranging from 0% to 46.1% [5]. *Strongyloides* infection is more prevalent among those who are socioeconomically disadvantaged, institutionalized, or residing in rural areas. Agricultural activities are often associated with this infection. The primary mode of transmission for *Strongyloides* is through contact with soil contaminated with *Strongyloides* larvae [6]. Therefore, activities that increase exposure to soil, such as walking barefoot, contact with human waste or sewage, and occupations that involve working with contaminated soil, like farming and coal mining, increase the risk of infection [7,8]. Much less commonly, it can be transmitted by ingestion of contaminated water [9].

Moreover, several studies have established a correlation between *Strongyloides* and human T-cell lymphotropic Virus-1 (HTLV-1) infection. These studies have demonstrated that individuals infected with HTLV-1 are more susceptible to *Strongyloides* infection and are more likely to develop severe cases of strongyloidiasis once infected [10].

Life Cycle

The *S. stercoralis* lifecycle alternates between free-living and parasitic cycles. After adult female worms lay their eggs in the duodenal mucosa, the resulting rhabditiform larvae are shed in feces. Warm, humid soil allows the larvae to molt into either infective filariform larvae or progress to rhabditiform larvae [11]. Larvae that molt into filariform larvae can infect humans via penetration of the skin and replicate asexually within the host. The larvae then enter the blood to travel to the lungs, where they ascend before reaching the GI tract [11]. 

**Figure 1:**
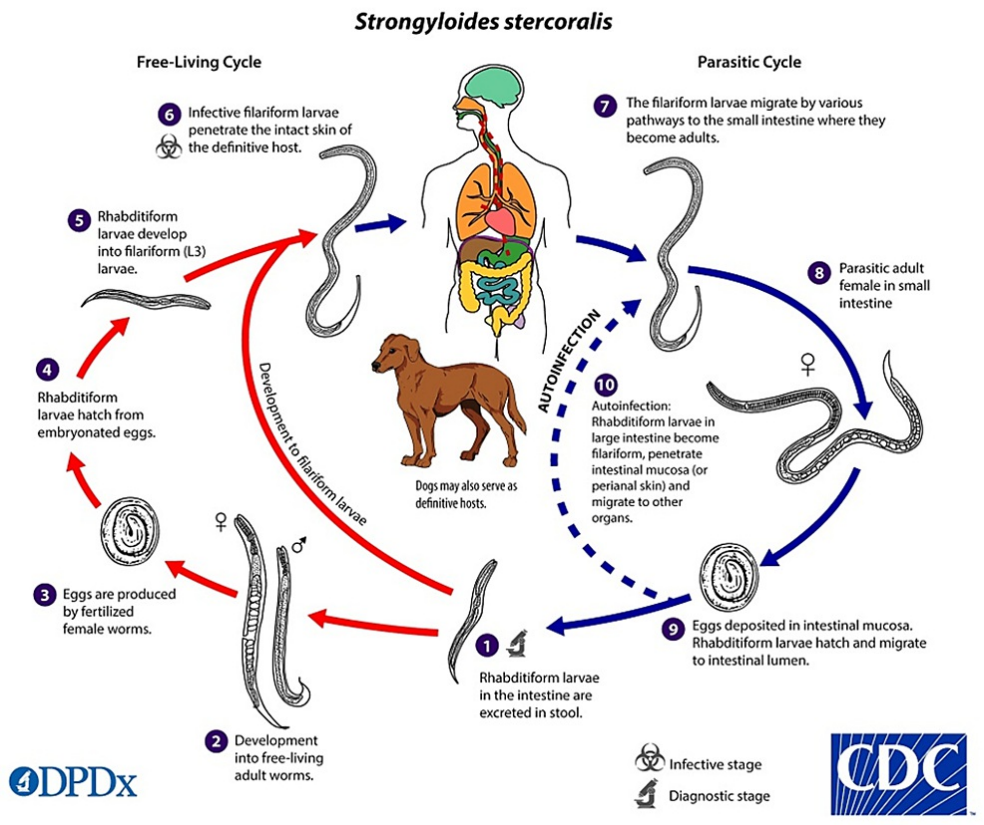
Life cycle of Strongyloides stercoralis Source: Center for Disease Control and Prevention (https://www.cdc.gov/dpdx/strongyloidiasis/index.html)

On the other hand, the larvae may progress through rhabditiform stages, in which they become free-living adults that infect hosts via ingestion of contaminated water sources and reproduce sexually. Both forms of larvae are capable of infection via ingestion of contaminated water (Figure 1).

**Table 1:** Clinical features of Strongyloidiasis

Acute Strongyloidiasis	Clinical Features
Constitutional	Fatigue, weakness, generalized pain, high eosinophil count
Skin manifestations	Pruritic rash/irritation, linear or petechial patterns
Pulmonary manifestations	Wheezing, shortness of breath, dryg cough, tracheal irritation, respiratory alkalosis, interstitial infiltrates on chest x-ray
Gastrointestinal manifestations	Abdominal tenderness, diminished bowel sounds, hypokalemia, anorexia, protein-losing enteropathy with peripheral edema, upper GI tract includes ulcerations, inflammation, necrosis, disruption of crypts, can mimic small bowel distention
Hyperinfection	Clinical features similar to acute but more severe including disseminated intravascular coagulation, renal failure, respiratory failure, gut translocation of bacteria resulting in bacteremia, low eosinophil count
CNS manifestations	CSF reveals aseptic meningitis
GI manifestations	Intestinal obstruction
Disseminated infection	Spreads beyond its normal migratory pathway without severity differences. May include lymph nodes, exocrine organs, heart, muscles, and kidneys.
Respiratory manifestations	Acute respiratory failure (can be mistaken for asthma exacerbation or pulmonary embolism
Gastrointestinal manifestations	Gram-negative or polymicrobial bacteremia
Chronic infection	Asymptomatic or mild symptoms
Gastrointestinal	Abdominal bloating, epigastric pain (especially with eating), sporadic vomiting, diarrhea, constipation, borborygmus, upper GI bleeding, ascites, chronic malabsorption, hepatic lesions
Skin manifestations	Larva currens of the buttock perineum, and thighs, hives or maculopapular eruptions
Other	Arthritis, cardiac arrhythmias, pseudopolyposis (on endoscopy), nephrotic syndrome, asthma

Pathogenesis

Once infected, patients may be acutely or chronically infected. The latter may present with or without symptoms. Such symptoms often follow the path of larval migration through the body. In certain patients, *S. stercoralis *may multiply without limit through autoinfection. Autoinfection occurs when rhabditiform larvae traveling within the GI tract transform into filariform larvae, allowing them to penetrate the gut wall or perianal skin and reinfect the host. This cycle permits the indefinite perpetuation of infection [1,11]. Autoinfection allows for hyperinfection, which is repeated re-infection. Autoinfection can also cause disseminated infection, which is the spread of the parasites to organs beyond the normal path. Hyperinfection and disseminated infection occur most frequently in immunocompromised or immunosuppressed patients [4,12].

Current research suggests that the immune response to *S. stercoralis* is mediated by CD4+ TH2 cells to prevent the uncontrolled replication of hyperinfection. The CD4+ response results in the secretion of pathogen-specific immunoglobulins. Therefore, infections, drugs, or disease states that suppress the TH2 response will put patients at a higher risk of either more severe or chronic *S. stercoralis* infection [13].

Clinical manifestations****

Acute

The presentation of *S. stercoralis* infection varies in onset, symptom types, severity, and duration of disease. Constitutional symptoms of strongyloidiasis may include fatigue, general pain, and weakness, but patients usually do not experience fever and chills [11]. Specific symptoms are based on the larval migration path within the human body, which includes the skin, lungs, and gastrointestinal tract [14,15].

Skin manifestations can include pruritic rash or irritation, especially at the site of penetration [11,14]. Specifically, there may be linear or petechial patterns on the skin created by larval migration [16]. Given that *S. stercoralis* enters the body through the skin, these symptoms typically occur early in the disease course.

Pulmonary manifestations can include wheezing, shortness of breath, dry cough, and possible tracheal irritation as the larvae travel from the skin to the lungs [14,15,17]. Respiratory alkalosis is commonly seen in strongyloidiasis. Radiologically, disruption of the pulmonary environment is evident as interstitial infiltrates that can be either localized or bilateral and widespread [11]. It is in the lungs that adult worms may be found on autopsy [18]. It is important to consider that a patient’s known medical conditions including asthma may mask pulmonary symptoms of strongyloidiasis. For example, one case study described a pregnant woman with a history of asthma and medical noncompliance presenting with a persistent cough and who was later discovered to have *S. stercoralis* larvae in her stool [19].

Strongyloidiasis presents most commonly without gastrointestinal symptoms [19]. In symptomatic cases, common findings include abdominal tenderness with diminished bowel sounds, hypokalemia, anorexia, and protein-losing enteropathy with resulting peripheral edema [20,21]. Upper gastrointestinal tract involvement of the esophagus, stomach, and small bowel are also common in symptomatic cases and include ulcerations, inflammation, necrosis, and disruption of crypts. Gastrointestinal involvement may be evident on abdominal imaging as small bowel distention with air-fluid levels or on radiological examination with CT [22,23].

Hyperinfection

Hyperinfection occurs due to the capacity of *S. stercoralis* for autoinfection, leading the parasitic burden to become extremely high in the skin, lungs, and gastrointestinal tract [24]. In contrast, disseminated infection occurs when the parasites disseminate out of the locations involved in the life cycle.

Strongyloidiasis hyperinfection syndrome includes the symptoms of common *S. stercoralis* infection along with more severe symptoms such as disseminated intravascular coagulation, renal failure, and respiratory failure due to the high parasitic burden [25]. Involvement of the CNS, evident as a spinal fluid examination revealing aseptic meningitis, is a common feature of hyperinfection [17]. Strongyloidiasis hyperinfection can also cause gut translocation of bacteria, resulting in bacteremia with gram-negative rods such as *E. coli *and gram-positive cocci such as *Streptococcus bovis* [26]. Intestinal obstruction is another known complication of S*. stercoralis* hyperinfection, as reported in at least two cases [15,24]. This complication can be fatal and is often seen in patients with other comorbidities of the lung [19]. Notably, although most cases of* S. stercoralis* infection are asymptomatic with eosinophilia being the only incidental finding, hyperinfection may instead present with low eosinophil counts [14,27].

Disseminated

Disseminated infections occur when *S. stercoralis *spreads beyond its normal migratory pathway but may not be associated with any difference in severity. Such tissues include lymph nodes, exocrine organs, the heart, muscles, and kidneys [28]. 

The primary risk factors associated with disseminated *Strongyloides* infection are immunosuppressive treatments, particularly corticosteroids, organ transplant, hematologic malignancy, and HTLV-1 infection [29]. These factors increase the likelihood of developing severe cases of strongyloidiasis.

Minor risk factors for disseminated infection include malnutrition, diabetes mellitus, chronic kidney disease, and alcoholism [30]. While these factors may not directly cause disseminated infection, they can weaken the immune system and make individuals more susceptible to severe cases of strongyloidiasis.

In cases of disseminated infection, the respiratory tract is the system most frequently affected outside the gastrointestinal tract. Acute respiratory failure secondary to the direct invasion of organs by the filariform larvae is often mistaken for asthma exacerbation or even pulmonary embolism.

Another common presentation of disseminated infection is gram-negative or polymicrobial bacteremia resulting from the migration of larvae through the bowel wall. Fever, gram-negative meningitis, peritonitis, and necrotizing jejunitis have also been reported in the literature as presentations of disseminated infection [30,31].

Hyperinfection and disseminated infection can also result in decades-long chronic infection and have significantly higher mortality rates in the range of 87.1%-100% [1,32]. Despite therapy, mortality with therapy can exceed 25% [27].

Chronic Strongyloidiasis

The majority of individuals who have chronic strongyloidiasis are asymptomatic or only experience mild symptoms. Most common are the gastrointestinal (GI) symptoms that include sensations of abdominal bloating, and epigastric pain that intensify with eating, sporadic vomiting, diarrhea, constipation, and borborygmus [33]. Given the nonspecific nature of these, it can be challenging to suspect strongyloidiasis unless accompanied by other indications or symptoms [34].

Larva currens, a serpiginous urticarial rash secondary to intradermal migration of *Strongyloides* larvae at 5-15 centimeters per hour is a commonly reported sign of chronic infection [33]. The buttock, perineum, and thighs are the most commonly involved sites. Repeated autoinfection is the cause of larva currens. Other dermatological indications of the disease include recurring rashes that appear as hives or maculopapular eruptions [35].

Some patients diagnosed with chronic strongyloidiasis experience arthritis and cardiac arrhythmias. Additionally, a few cases of endoscopic examinations revealed pathological features akin to pseudopolyposis.

Nephrotic syndrome, massive upper GI bleeding, ascites, chronic malabsorption, hepatic lesions, arthritis in individuals who are HLA-B27 positive, and asthma are some of the less common manifestations [36-42].

Strongyloidiasis in pregnancy

Pregnancy can depress or otherwise alter the immune system, leading to a decreased immune response to parasitic infections [14]. Physiological changes that occur during pregnancy include alterations to the maternal immune system [43]. These changes may include a decreased ability to fight parasitic and viral infections, including* S. stercoralis* [43,44]. Although it has been suggested that some parasitic infections during pregnancy lead to placental inflammation and thus negatively affect the fetus, there is no such evidence regarding *S. stercoralis* in particular [45]. In some pregnant patient cases, *S. stercoralis* infection and symptoms can be associated with coinfection with HTLV-1 [12,15]. HTLV-1, as previously stated, puts patients in an immunosuppressed state [12]. Additionally, glucocorticoids are a common drug used in premature pregnancies.

There is no corroborated evidence of *S. stercoralis* infection in pregnant females resulting in adverse outcomes in the mother, fetus, or childhood outcomes. This is mainly due to a gap in knowledge and a lack of literature on the subject [19,44].

Breast milk concentration of the ivermectin after a single oral dose is within the WHO threshold for safe breastfeeding [46]. Its concentration in breastmilk after topical use is not well studied, but it is considered safe since systemic absorption of ivermectin following topical use is low compared to oral administration, provided that the breast area is free of contamination while breastfeeding [47].

Strongyloidiasis in transplant patients

Immunocompromised individuals, such as transplant patients, are more severely affected by infection from* S. stercoralis* and have a worse prognosis [48]. In these individuals, due to an exaggerated autoinfection, hyperinfection and dissemination can also occur [49]. This has proven to be highly fatal, as mortality rates are approaching 80% [50]. It has been reported in lung, heart, liver, and combined heart and kidney transplant recipients. Pre-transplant exposure in recipients was more recurring in endemic areas [25,51-58]. Low and middle-income countries are also endemic to strongyloidiasis [2,59]. The pre-transplant assessment of cases should involve a thorough examination of their medical history, including travel history, and a comprehensive physical examination [58]. Steroids given to recipients deplete T-cells and place the host at an increased risk of reactivation. Reactivation of prior chronic infection, or from the donor, are the two main causes of post-transplant infection [50]. Screening before transplant is essential in immigrants from countries that are endemic to strongyloidiasis and recipients with marked eosinophilia [49,58]. Cases that exhibit non-specific GI or cardiopulmonary symptoms, or who develop unexplained sepsis due to gram-negative bacteria, should be evaluated for strongyloidiasis [50].

Kidney transplant recipients are at a higher risk of developing severe infection that commonly manifests as GI or respiratory symptoms, or both. Epigastric pain, nausea, vomiting, diarrhea, and loss of appetite are common GI symptoms. Dyspnea, cough, hemoptysis, and bronchospasm are the respiratory symptoms that cases present with. Cases may or may not also present with fever and eosinophilia. Both, respiratory failure and the simultaneous presence of bacteremia were identified as factors that independently predicted mortality. Cases infected within the first three months post-organ transplantation exhibited significantly higher respiratory failure and 30-day mortality rates. Receiving an organ transplant from a deceased donor, having a prior bacterial infection, and the cumulative dose of corticosteroids were identified as a few independent risk factors. A few complications in immunosuppressed hosts are respiratory failure, septic shock, gram-negative bacillary infections, bacteremia, meningitis, gastrointestinal bleeding, paralytic ileus, and malabsorption. Reported outcomes are death and significant graft dysfunction. Coinfection with *Cytomegalovirus*, oral or esophageal candidiasis, herpetic esophagitis, and pulmonary tuberculosis have been seen [60].

Cyclosporine, an immunosuppressant, has been proven to be toxic to this nematode and is associated with a reduced risk [50]. Albendazole, thiabendazole, and ivermectin are used as prophylactic agents to prevent infection. These also delay the disease occurrence and lower the mortality rate [60]. Management of strongyloidiasis relies on screening and a high degree of suspicion in at-risk cases. RT-PCR is an important diagnostic tool that helps in screening donors and early diagnosis of recipients from infected donors, which can lead to timely treatment and prevention [50]. Treatment of the majority of severe cases of *S. stercoralis* consisted of a combination of drugs, generally ivermectin and albendazole [60].

Strongyloidiasis in COVID-19 patients

In our literature search, a total of six cases of *Strongyloides* hyperinfection in COVID-19 were reported. Two patients were administered dexamethasone and tocilizumab and the rest of the four COVID-19 patients were treated with steroids alone [61,62]. It has been observed that even short courses of corticosteroids in six days can trigger this condition [28]. To prevent morbidity and mortality from *Strongyloides* hyperinfection, it is crucial to identify and treat occult or subclinical infections in the immigrant population hospitalized for COVID-19. However, the sensitivity and specificity of *Strongyloides* tests pose a challenge, and diagnostic testing is often not readily available or timely. In such situations, the use of presumptive ivermectin treatment may be reasonable in selected cases of COVID-19 [63].

Diagnosis

Prompt suspicion of strongyloidiasis in patients with suggestive signs and symptoms, eosinophilia, and a history of walking barefoot in an endemic area is key to diagnosis. Eosinophilia is more common with *S. stercoralis* infection than with other helminths [64]. It is usually mild, intermittent, and nonspecific with a poor positive predictive value of 15% [65]. Therefore, eosinophilia alone is of limited use in diagnosis. Elevated total IgE along with eosinophilia was found in 38%-59% of the cases. Eosinophilia is often absent in cases of disseminated infection [30].

Serology and detection of larvae in stool are the most commonly used methods to diagnose strongyloidiasis [66]. Serology demonstrated a sensitivity and negative predictive value of 100% with a specificity of 97% and a positive predictive value of 67%. On the other hand, microscopic examination of stools showed a significantly lower sensitivity of 45% with a specificity and positive predictive value of 100%. Additionally, the negative predictive value of stool examination was high at 96% [67]. The fecal tests involve collecting a stool sample and examining the contents microscopically for *S. stercoralis *larvae. This approach lacks sensitivity due to the variability of larval excretion between patients, though an increase in the number of stool tests done on one patient will increase the likelihood of detecting larvae and hence the sensitivity [1,66]. The collection of seven stool samples and the application of specialized testing techniques is the recommended gold standard by the CDC [68]. Another method for improving the sensitivity of fecal tests is the agar plate method, which allows the larvae to crawl across the agar plate and create a path of bacterial growth in their wake. The agar plate culture method is 96% sensitive but is laborious and time-consuming [69].

Serological testing is considered the most sensitive method for diagnosing strongyloidiasis [66]. A recent study of the sensitivities and specificities of six different serological studies found them all to be > 82.6% sensitive and > 70.8%-97.8% specific [70]. It is important to note, however, that data from sensitivity and specificity trials are affected by the lack of a reliable gold standard for the diagnosis of strongyloidiasis [66]. Serological methods are better than fecal diagnosis to estimate the prevalence [71].

Differential diagnosis

When assessing a patient for suspected strongyloidiasis, it is important to consider other parasitic conditions such as acute schistosomiasis (also known as Katayama fever), ascariasis, amebiasis, human hookworm infection (caused by *Ancylostoma duodenale* or *Necator americanus*), and zoonotic infections (such as *S. mesopotamia,* *S. procyonis, Ancylostoma braziliensis, *or *A. caninum*) [72].

However, it is also important to keep in mind nonparasitic conditions that may present similarly, including polyarteritis nodosa, systemic lupus erythematosus, contact dermatitis, erythema annular centrifugum, scabies, urticaria, anaphylaxis, drug reactions, Henoch-Schönlein purpura, eosinophilic or bacterial gastroenteritis, malabsorption, malnutrition, upper and lower gastrointestinal bleeding, peptic ulcer disease, transient pulmonary eosinophilic syndrome, pneumonia, meningitis, and sepsis and/or septic shock, and other conditions associated with eosinophilia [73].

It is worth noting that *Strongyloides* colitis, while easily treatable, can mimic ulcerative colitis and potentially lead to fatal outcomes [74]. Therefore, clinicians should maintain a high level of suspicion and be aware of the gastrointestinal similarities between the two conditions.

Treatment****

In clinical practice, treatment of strongyloidiasis is more prone to failure because of autoinfection and impairment of host immunity, therefore, it can be challenging. Since autoinfection can occur in two to three weeks, dosage should be repeated in the two- to three-week interval to make the treatment certain [75]. 

The most commonly used drugs for the treatment of strongyloidiasis are benzimidazoles like albendazole thiabendazole and ivermectin. Ivermectin is a highly potent, broad-spectrum anthelmintic drug [49]. It is a semi-synthetic derivative of a family of macrocyclic lactones called the avermectins. It acts by causing an influx of chloride ions through the cell membrane of the parasite by activation of specific ivermectin-sensitive ion channels. The resultant hyperpolarization leads to muscle paralysis. Oral ivermectin is reported to have superior cure rates compared to other anthelmintics (albendazole, thiabendazole) with a better side effect profile [76]. Studies have reported it to be effective in reducing seroprevalence in highly endemic areas [77]. It is not only the adult stage but also the larval form of *S. stercoralis*. These features make oral ivermectin the drug of choice for strongyloidiasis [32,76,78]. Rectal or subcutaneous administration of ivermectin is an alternative in cases of severe disease or when oral intake is not possible [25]. The bioavailability of the drug can be increased by giving it with food. Cytochrome P450 and CYP3A4 metabolize this drug in the liver and is minimally excreted in the urine [79]. Concomitant Loa loa infection is a contraindication of ivermectin. Abnormal liver function tests, itching, and neurological symptoms are a few side effects reported with ivermectin [75]. A single dose of ivermectin 200 microgram/kilogram is shown to be effective in the treatment of non-disseminated infection [80]. For disseminated infection with larvae in sputum and stool, it is recommended to repeat treatment every 15 days until stool tests are negative for larvae and then 1 more treatment cycle [28].

As of now, hyperinfection is a medical emergency requiring rapid initiation of ivermectin treatment for a minimum of two weeks, usually until two full weeks have passed with negative tests on stool samples [11]. Efforts should also be taken to reduce the immunosuppressed state of patients when possible (e.g., reducing the dose of immunosuppressive drugs during the course of *S. stercoralis *treatment).

The use of ivermectin in human pregnancy is not well studied, and its teratogenic risks are not known [81]. Due to a lack of proper studies in pregnant women, ivermectin is classified as a category C drug by the US Food and Drug Administration (FDA). Ivermectin has known teratogenic side effects in rats and rabbits at doses 10 times higher than the human therapeutic dose. There are reports of ivermectin use in pregnancy with no adverse effects on the mother or the fetus [82]. Based on mass drug administration (MDA) campaigns in African countries, pregnant females were exposed to ivermectin. However, according to limited literature, no maternal or fetal side effects were noted [83]. The decision to take ivermectin in pregnancy should be based on risk versus benefit analysis. For pregnant females considered immune deficient, a dose of oral ivermectin is typically 200 mcg/kg/day, which should be continued until two weeks of daily stool analysis come back negative [15]. It is important to note that the risk of hyperinfection, disseminated disease, septic shock, and death is increased when corticosteroids are administered to improve fetal lung maturity in premature pregnancies [12,15]. There is a need for not only research into ivermectin use in pregnancy but also its use in hyperinfection or disseminated infection in pregnant women.

Another drug, moxidectin, from the same family as ivermectin, binds to the glutamate-gated chloride channels and γ-aminobutyric acid receptors. This leads to increased permeability, and therefore, muscle paralysis [84]. Moxidectin has been shown to have similar cure rates to ivermectin for strongyloidiasis, as shown by a phase IIa dose-finding study. Moxidectin is well tolerated and efficient at a dose of 4-12 mg for treating the infection [85]. A dose of 8 mg is suitable, which is the same as the dose registered for onchocerciasis [86].

Albendazole treatment regimens are also widely explored for* S. stercoralis.* Albendazole acts by selectively binding to beta tubulin in parasitic worms leading to their immobilization and death. It is more effective than mebendazole due to its better absorption [87]. A recommended treatment dosage is of 400 mg/day in divided doses for three days which is repeated one week later [88].

An RCT compared two different high-dose regimens of albendazole and recommended giving albendazole at 800 mg/day twice daily for three consecutive days rather than giving the same dose for five consecutive days [89]. A clinical trial testing the efficacy of albendazole showed that a high cure rate of 86% was obtained with a dosage of 16 mg/kg/day for three days and repeated once after a fortnight [90]. It is usually well tolerated; however, it has some common side effects like gastrointestinal upset, dizziness, headaches, and hair loss. Some of the rare but severe side effects include bone marrow suppression, pancytopenia, granulocytopenia, sudden worsening of neurocysticercosis, hypersensitivity reactions, rash, erythema multiforme, and Stevens-Johnson syndrome [87].

Prevention

At this time, there is no global strategy to mitigate* S. stercoralis *in endemic regions [4]. Prevention of strongyloidiasis is attempted through decreasing environmental risk factors, increasing awareness, and administration of deworming medications [44].

Possible determinants and risk factors for infection include lack of footwear, infected family members, poor sanitation, and HTLV status [24,44]. Increasing Strongyloidiasis screening through ELISA and/or serology can help with early detection. Screening for HTLV-1 status would be helpful in possible relapse [24,91].

Increasing awareness begins with having accurate knowledge about the prevalence of strongyloidiasis. The commonly cited statistic of 30-100 million infections is a gross underestimation of the newly estimated 613.9 million cases in existence, which translates to 8.1% of the world’s population [4]. Finally, oral ivermectin administration to communities as a deworming method could help prevent future infections and prevent the continued fecal-oral spread of *S. stercoralis* in endemic areas to decrease the risk of infection [44].

## Conclusions

*S. stercoralis* infection results in clinical practice challenges, including diagnostic complexities, and a broad spectrum of disease presentations depending on the host’s immunity. In a normal host, it can cause acute infection or chronic asymptomatic infection. In contrast, poor host immunity, high parasite burden from autoinfection, and concurrent bacterial and fungal infection lead to high mortality in hyperinfection and disseminated strongyloidiasis. The diagnosis of acute and chronic strongyloidiasis is difficult because of the non-specific presentations and low parasite load. All available investigations have limited sensitivity. Thus, the combination of parasitological and serological methods is recommended. Prompt suspicion and screening of high-risk groups, particularly individuals with compromised immune systems or those taking immunosuppressive medications like corticosteroids, COVID-19 patients with risk factors is crucial to prevent the severe consequences of hyperinfection syndrome. In cases where screening is not possible, it is highly recommended to provide immediate treatment to these patients.

The gold standard regimen for the treatment of strongyloidiasis is a single dose of ivermectin. Moxidectin has the potential to become the drug of choice in the future. Increases in traveling and migration, as well as advances in immunosuppressive treatment, particularly during the COVID-19 pandemic, have raised the impact and awareness of strongyloidiasis. Active surveillance and further research regarding diagnostic techniques are required to reveal the real burden of this under-reported disease.
